# A matter of frailty: the modified Subdural Hematoma in the Elderly (mSHE) score

**DOI:** 10.1007/s10143-021-01586-2

**Published:** 2021-07-06

**Authors:** Silvia Hernández-Durán, Daniel Behme, Veit Rohde, Christian von der Brelie

**Affiliations:** 1 Department of Neurosurgery, Universitätsmedizin Göttingen, Robert-Koch-Str. 40, 37075 Göttingen, Germany; 2 Institute for Diagnostic and Interventional Neuroradiology, Universitätsmedizin Göttingen, Robert-Koch-Str. 40, 37075 Göttingen, Germany; 3 University Clinic for Neuroradiology, Universitätsklinikum Magdeburg A. Ö. R, Leipziger Str. 44, 39120 Magdeburg, Germany

**Keywords:** Chronic subdural hematoma, Elderly, Frailty, Prediction models

## Abstract

The Subdural Hematoma in the Elderly (SHE) score was developed as a model to predict 30-day mortality from acute, chronic, and mixed subdural hematoma in the elderly population after minor or no trauma. Emerging evidence suggests frailty to be predictive of mortality and morbidity in the elderly. In this study, we aim to externally validate the SHE for chronic subdural hematoma (CSDH) alone, and we hypothesize that the incorporation of frailty into the SHE may increase its predictive power. A retrospective cohort of elderly patients with CSDH after minor or no trauma being treated at our institution was evaluated with the SHE. Thirty-day mortality and outcome were documented. Patients were assessed with the Clinical Frailty Scale (CFS), which was incorporated into a modified SHE (mSHE). Both the SHE and the mSHE were then assessed in their predictive powers through receiver operating characteristic statistics. We included 168 patients. Most (n = 124, 74%) had a favorable outcome at 30 days. Mortality was low at n = 7, 4%. The SHE failed to predict mortality (AUC = .564, p = .565). Contrarily, the mSHE performed well in both mortality (AUC = .749, p = .026) and outcome (AUC = .862, p < .001). A threshold of mSHE = 3 is predictive of mortality with a sensitivity of 50% and a specificity of 75% and of poor outcome with a sensitivity of 88% and a specificity of 64%. Frailty should be routinely evaluated in elderly individuals, as it can predict outcome and mortality, providing the possibility for medical, surgical, nutritional, cognitive, and physical exercise interventions.

## Introduction

Globally, there has been a steady increase in the elderly population, and current projections estimate a total of > 1.5 billion individuals aged > 65 years by 2050 [1]. For neurosurgeons, this demographic trend presupposes a concomitant, continuous increase in patients suffering from a chronic subdural hematoma (CSDH), a condition traditionally associated with old age [2]. Epidemiologic studies have shown the incidence of CSDH to increase from 3.4/100,000/year in individuals < 65 years, to up to 58–127/100,000/year in the elderly [3]. Thus, CSDH in the elderly will become an even more prevalent pathology in neurosurgery in future decades.

Geriatric patients present unique challenges, as studies have shown age to be a predictor of poor outcome and mortality, irrespective of surgical discipline and pathology treated [4, 5]. Careful and comprehensive counseling of elderly patients and their families thus becomes pivotal in clinical decision-making. When deciding which patients should undergo further treatment of their CSDH, proper assessment of their prognosis is key. To aid clinicians in their decision-making and advising, the Subdural Hematoma in the Elderly (SHE) score was published in 2019 as a model to predict 30-day mortality from a subdural hematoma in the elderly population after minor or no trauma [6].

To develop this score, patients suffering from acute SDH (ASDH), chronic SDH (CSDH), and mixed-acuity SDH (MASDH) from a consecutive retrospective cohort were analyzed [7]. Admission Glasgow Coma Scale (GCS), age, and hematoma volume were identified as predictive variables. Emerging evidence however suggests that the relationship between age and outcome is not necessarily clear-cut and linear. Frailty, an age-related cumulative decline in multiple physiological systems, has been shown to be a better predictor of mortality and morbidity than chronological age alone in multiple conditions [4]. Furthermore, ASDH and CSDH are distinct clinical entities with different underlying pathophysiologies and clinical courses: e.g., CSDH usually presents with subtle symptoms not necessarily reflected by GCS [8], while ASDH usually leads to rapid neurologic deterioration. In this study, we aim to externally validate the SHE for CSDH alone, excluding ASDH and MASDH. Furthermore, we hypothesize that the incorporation of frailty assessment into the SHE can increase its predictive power for mortality and outcome in elderly patients with CSDH.

## Materials and methods

### Patient population, inclusion, and exclusion criteria

We conducted a retrospective study of consecutive elderly patients admitted to our center with CSDH from January 2015 to September 2019. Due to the retrospective nature of the study, no informed consent was necessary. The study was carried out in accordance with our institutional protocols and the 1964 Helsinki declaration (IRB 15/1/21). In analogy to the methods used by Alford et al. [6], patients were considered to be “elderly” if they were > 65 years. As stipulated by the SHE, only patients with a history of minor or no trauma were included. Patients who had sustained high-velocity trauma, such as a fall from a height > 3 m, motor-vehicle accident, or pedestrian struck were excluded. Baseline demographic characteristics, GCS at admission, and hematoma volume, as calculated by the A*B*C/2 formula, were recorded and scored according to the SHE. Additionally, presenting symptoms were evaluated by means of the Markwalder Grading System (MGS) for CSDH. All patients included were initially treated by means of minimally invasive twist-drill-craniostomy (TDC) under local anesthesia, as described by Reinges et al. [9].

### Frailty assessment and the modified SHE

To test our hypothesis, we assessed patients by means of the Canadian Study of Health and Aging (CSHA) Clinical Frailty Scale (CFS) [10, 11] (Table 1), a tool that has been validated for retrospective application [12]. We chose this scale because it was shown to correlate with outcome in a logistic regression analysis in a retrospective series of 211 CSDH elderly patients [13]. Thus, we maintained the same methodology for variable selection employed by Alford et al. in their original publication [6], namely the Prognosis Research Strategy (PROGRESS) and the Transparent Reporting of a multivariate prediction model for Individual Prognosis or Diagnosis (TRIPOD) [14].

**Table 1:** CFS for frailty evaluation^10,11^

Score	Clinical description
1	Very fit—People who are robust, active, energetic, and motivated. These people commonly exercise regularly. They are among the fittest for their age
2	Well—People who have no active disease symptoms but are less fit than category 1. Often, they exercise or are very active occasionally, e.g., seasonally
3	Managing well—People whose medical problems are well controlled but are not regularly active beyond routine walking
4	Vulnerable—While not dependent on others for daily help, often symptoms limit activities. A common complaint is being “slowed up” and/or being tired during the day
5	Mildly frail—These people often have more evident slowing and need help in high order activities of daily life (finances, transportation, heavy housework, medications). Typically, mild frailty progressively impairs shopping and walking outside alone, meal preparation, and housework
6	Moderately frail—People need help with all outside activities and with keeping house. Inside, they often have problems with stairs and need help with bathing and might need minimal assistance (cuing, standby) with dressing
7	Severely frail—Completely dependent for personal care, from whatever cause (physical or cognitive). Even so, they seem stable and not at high risk of dying (within approximately 6 months)
8	Very severely frail—Completely dependent, approaching the end of life. Typically, they could not recover even from a minor illness
9	Terminally ill—Approaching the end of life. This category applies to people with a life expectancy < 6 months, who are not otherwise evidently frail

We included the CFS in the modified SHE (mSHE), allotting 0 points for CFS 1–3, 1 point for CFS 4–5, and 2 points for CFS 6–9. These thresholds were derived from retrospective validation studies, in which the CFS was evaluated in its ability to identify patients who would benefit from intensive care management [15] or resuscitation after cardiac arrest [16]. Table 2 summarizes the components of the SHE and the mSHE.

**Table 2:** Criteria contained in the SHE and mSHE

Criterion	Points
Age	
< 80 years	0
≥ 80 years	1
GCS at admission	
13–15	0
5–12	1
3–4	2
Hematoma volume	
< 50 ml	0
≥ 50 ml	1
CFS at admission*	
1–3	0
4–5	1
6–9	2

^*^Denotes criteria added in the mSHE

### Outcome

Since the SHE was developed to predict 30-day mortality in SDH patients, we collected data on mortality in our patient population as the primary endpoint of this study. Furthermore, we assessed patient outcome at 30 days by means of the Glasgow Outcome Scale (GOS) as a secondary endpoint. The outcome was dichotomized in “good” for GOS ≥ 4 and “poor” for GOS ≤ 3.

### Statistical analysis

Receiver operating characteristic (ROC) curves were calculated for the SHE and the mSHE, for both mortality and dichotomized outcome (“good”/”poor”) in CSDH. Statistical significance was assumed at p < 0.05. Youden’s index was used to determine the best cutoff values in the SHE and the mSHE for the prediction of mortality and outcome. Analysis was performed using IBM® SPSS® v. 21.

## Results

A total of 168 patients were included. Of these, n = 112, 67% were males. Median age was 79 years (interquartile range (IQR) 10.75). Most patients, n = 103, 61%, were on anticoagulants and/or antiaggregants. Clinically, n = 60, 36% presented with mild symptoms (MGS 0–1), n = 63, 38% with moderate symptoms (MGS 2) and n = 45, 27% with severe symptoms (MGS 3–4). Median hematoma volume was 121 ml (IQR 79).

**Fig. 1:**
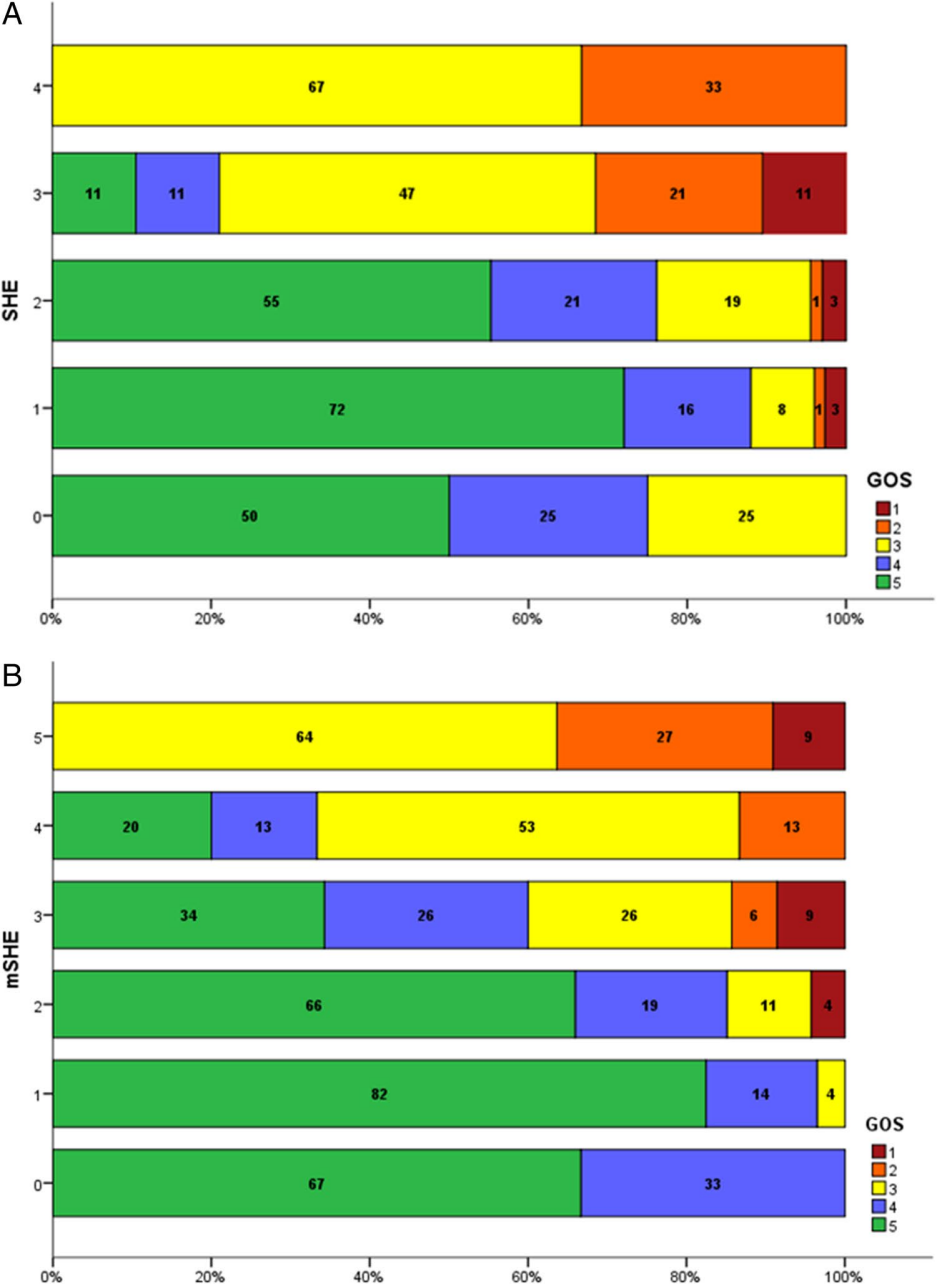
**A** GOS stratified by SHE score. **B** GOS stratified by mSHE score

Most patients (n = 124, 74%) had a favorable outcome of GOS 4–5 at 30 days. Mortality was low at n = 7, 4% in the entire cohort. As illustrated in Fig. 1, good outcome was observed in 75% of patients with SHE = 0, 88% with SHE = 1, 76% with SHE = 2, 21% in SHE = 3, and 0% with SHE = 4. For mSHE, good outcome was 100% at mSHE = 0, 97% at mSHE = 1, 85% at mSHE = 2, 60% at mSHE = 3, 33% at mSHE = 4, and 0% at mSHE = 5. Patients with SHE = 4 had 0% mortality. The distribution of outcome according to SHE/mSHE is summarized in Table 3.

**Table 3:** Patients stratified according to GOS and SHE/mSHE

Score		GOS				
		1	2	3	4	5
SHE	0	0	0	1	1	2
	1	2	1	6	12	54
	2	2	1	13	14	37
	3	2	4	9	2	2
	4	0	1	2	0	0
mSHE	0	0	0	0	1	2
	1	0	0	2	8	47
	2	2	0	5	9	31
	3	3	2	9	9	12
	4	0	2	8	2	3
	5	1	3	7	0	0
	6	0	0	0	0	0

**Fig. 2:**
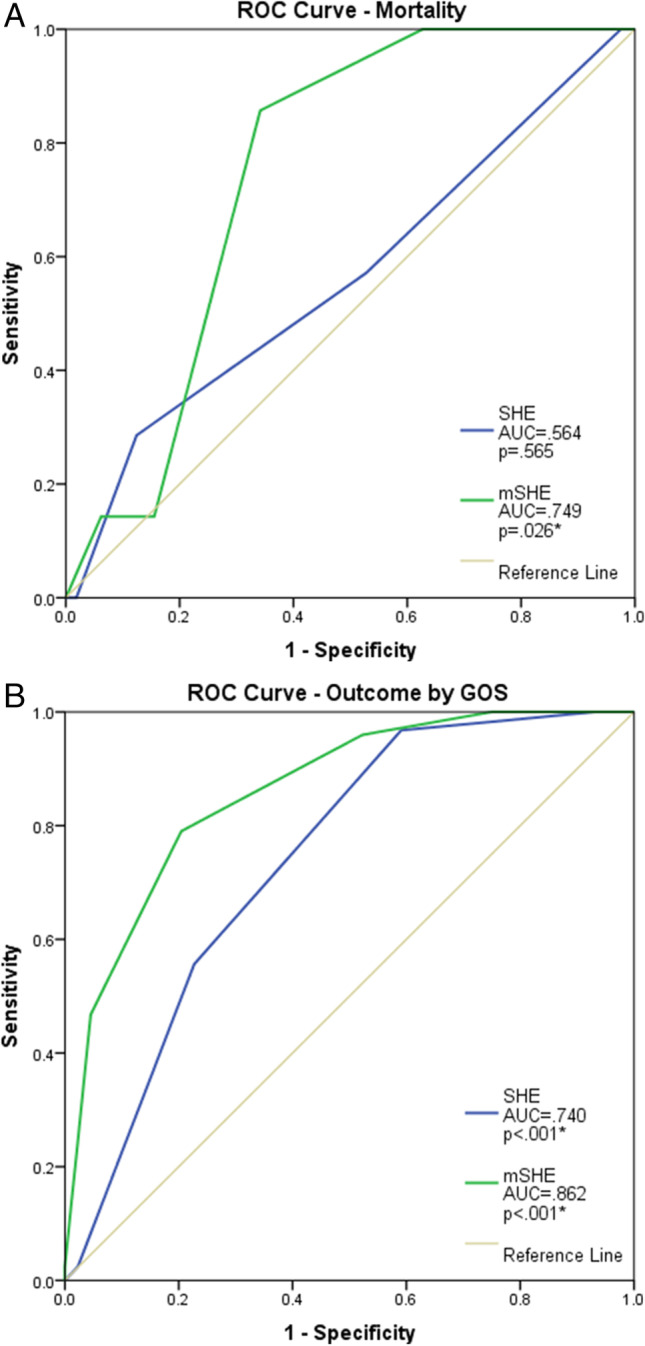
**A** ROC curve for a 30-day mortality prediction of both the SHE (blue) and mSHE (green). **B** ROC curve for a 30-day outcome prediction, defined by GOS, of both the SHE (blue) and mSHE (green)

As illustrated in Fig. 2, the SHE failed to predict mortality in our patient cohort (AUC = 0.564, p = 0.565), but it was a sensitive prognostic tool for outcome, as objectivized by GOS (AUC = 0.740, p < 0.001*). On the contrary, the mSHE performed well in both mortality (AUC = 0.749, p = 0.026*) and outcome prediction (AUC = 0.862, p < 0.001*). A threshold of mSHE = 3 is predictive of mortality with a sensitivity of 50% and a specificity of 75%; the same threshold is predictive of poor outcome with a sensitivity of 88% and a specificity of 64%.

## Discussion

In our cohort, the SHE was not a statistically significant model to predict 30-day mortality in CSDH. In the original publication by Alford et al. [6], the score also performed the worst in the CSDH subgroup, with an AUC of 0.8, compared to an AUC of 0.941 in ASDH. With almost twice as many patients with CSDH than the original cohort, 168 vs. 89, we could not validate the SHE for 30-day mortality prediction in CSDH. Thus, we recommend caution when relying on this model alone to guide clinical decision-making and counseling in patients with CSDH after no or minor trauma.

The authors postulate that their inability to validate the SHE was due to a methodological limitation of the original work, namely the pooling of patients with ASDH, MASDH, and CSDH from a retrospective study by Kuhn et al. [7]. In this study, mortality predictors from a multivariate analysis in the ASDH cohort were different from those in CSDH. GCS on admission, contusion volume, and age ≥ 80 years were predictive of mortality in ASDH. In the CSDH cohort, only GCS was associated with increased mortality. Evidently, the SHE relies on predictors more strongly associated with ASDH and obviates potential additional factors exclusively related to CSDH.

The observed differences in predictive factors in the study by Kuhn et al. [7] can be at least partially explained by the differing pathophysiologies of the hematoma types. On the one hand, ASDH is often associated with a rapid neurological decompensation and deterioration, due to the pooling of blood in the subdural space after tears in bridging veins or, in the majority, superficial cortical arteries, classically resulting from traumatic brain injury [17]. In the elderly, these tears can, and often do, result from minor trauma, such as falls while walking or from bed [18].

CDSH, on the other hand, is associated with neuroinflammatory processes. While the initial insult might be minor trauma, such as in ASDH, an inflammatory cascade with accompanying neo-angiogenesis, fibrinolysis, and excessive fluid exudation from newly formed membranes following an oncotic gradient characterizes this condition and explains its latency in clinical presentation and tendency to recurrence [19, 20]. Patients thus present with more subtle neurological symptoms [8, 21], and the natural history of this condition is often insidious [3]. Nevertheless, CSDH in the elderly has been termed a “sentinel health event,” heralding underlying systemic pathology in this patient population [2].

Notably, the mortality rate in ASDH in the elderly can be up to 60% [17, 18, 22]. In contrast, the mortality rate in CSDH in geriatric populations is almost half, at up to 32% [23, 24]. While mortality in ASDH can be attributed to the underlying brain injury and increased intracranial pressure [17, 18], mortality in CSDH varies greatly in its causes. Overall in-hospital mortality during index admission has been reported at 16% due to complications such as rebleeding, respiratory failure, and dementia; this rate rises to up to 32% at 6 months, with iatrogenic complications such as pulmonary embolism, stroke, or myocardial infarction taking on a more predominant role due to the discontinuation of anticoagulants in a highly comorbid population [25]. These greatly differing mortality rates provide further explanation as to why pooling patients with these two separate clinical entities for prediction modeling might not be advisable. Nonetheless, among neurosurgeons, CSDH is generally recognized as a benign condition and underestimation of fatality risk is common.

Another potential reason for our inability to externally validate the SHE for mortality prediction in CSDH might lie in the differing treatment algorithms. At our center, all patients with CSDH undergo TDC under local anesthesia as first-line surgical treatment [9]. In the original work by Kuhn et al. [7], on which the SHE was based, patients were treated by means of craniotomies, burr-hole evacuation, and minimally invasive techniques. A detailed subgroup analysis based on surgical techniques was not performed. Studies have failed to prove the superiority of one surgical technique over another, and medical management remains controversial [2, 26, 27]. In a systematic review by Ivamoto et al. [28], TDC and burr-hole evacuation were found to be equivalent. In a meta-analysis by Weigel et al. [29], all surgical techniques were found to have similar mortality (2–4%), but craniotomy was shown to have significantly higher morbidity than TDC. Seizures, empyema, pneumonia, and other medical complications more often ensue after more invasive surgical procedures [30]. Nevertheless, it is well established that geriatric patients have an increased risk for adverse outcomes when undergoing general anesthesia due to their comorbidities, polypharmacy, and decreased physiological reserve [5, 31]. Thus, the outcome in elderly patients undergoing treatment for CSDH can be greatly influenced by surgical and anesthetic techniques, which is not accounted for in the study by Kuhn et al. [7], and thus by the SHE. Due to our institutional protocols dictating TDC as first-line treatment for CSDH, our study population is more homogeneous, thus removing this potential confounder when evaluating scoring systems.

A main result of our study is that by incorporating frailty assessment into the SHE, we were able to increase its accuracy in mortality prediction in our patient cohort. Frailty is a geriatric syndrome characterized by decreased functional reserve and increased vulnerability against stressors [32]. It results from the cumulative decline of many physiological systems during a lifetime, and it is not necessarily dependent on other distinct comorbidities; it rather reflects their impact on the organism [12]. A study by Shimizu et al. [13] on 211 elderly patients with CSDH showed that those with frailty, objectivized by means of the CFS, had a poorer prognosis than those without. A systematic review of the literature on ASDH in the elderly [33] identified the presence of comorbidities to influence the outcome but also evinced the lack of literature objectively assessing comorbidities and frailty with validated instruments. While the evidence is only beginning to emerge on the importance of frailty assessment in CSDH, recent studies have illustrated how it can aid clinicians in prognostication in other neurosurgical pathologies, including glioblastoma [34], meningioma [35], and other primary central nervous system tumors [36]. Thus, the inclusion of this additional criterion into prognostication tools in CSDH appears warranted.

Alford et al. [6] suggest the SHE might be superior to other existing prediction models for SDH [37] thanks to the simplicity and readily availability of its components. The CFS is an easy-to-use scale, amply validated [10, 38] in multiple settings, both in medicine and surgery, so that the authors believe that its incorporation into the SHE does not make it more cumbersome.

Importantly, frailty is not an irreversible pathology, and it can be improved upon. A systematic review of randomized controlled trials showed that physical exercise interventions in frail elderly individuals can improve their outcomes [39]. Additionally, nutritional and cognitive interventions can reverse frailty [40]. Therefore, frailty assessment provides a tremendous opportunity for risk stratification and the incorporation of early, aggressive rehabilitation programs to optimize patient outcome. Scoring systems and clinical prediction models are critical to inform and counsel patients and their families. With the mSHE, the authors believe that clinicians can guide patients not only about CSDH, but also about the necessary rehabilitation interventions to recover from it.

### Limitations

This study has several limitations due to its retrospective nature. The data obtained from medical records might be inaccurate, especially when relying on them to extract the degree of frailty and judging on the outcome using GOS in a retrospective manner. In particular, due to the insidious nature of CSDH, the degree of frailty might have been misjudged, for CSDH might have had an unrecognized effect on the patient’s independence and physiological reserve before neurosurgical consultation. Finally, we did not assess the social network of the patients, which might have had an impact on their outcome.

## Conclusion

We failed to externally validate the SHE for mortality prognostication in elderly individuals suffering from CSDH. Thus, the authors warrant caution when relying on this model alone for patient counseling. By incorporating an objective, easy-to-perform clinical frailty assessment into the SHE, we were able to increase its performance and predict both mortality and outcome in geriatric patients with CSDH. Frailty should therefore be routinely evaluated in elderly individuals, for it can aid clinicians, caregivers, and patients develop a holistic therapeutic approach, including medical, surgical, nutritional, cognitive, and physical exercise interventions.

## Data Availability

Pertinent data are presented in the manuscript. Raw data can be made available at request pseudonymized.

## Code availability

N/A.
